# NK Cells in Ascites From Liver Disease Patients Display a Particular Phenotype and Take Part in Antibacterial Immune Response

**DOI:** 10.3389/fimmu.2019.01838

**Published:** 2019-08-07

**Authors:** Philipp Lutz, Hannah C. Jeffery, Nicholas Jones, Jane Birtwistle, Benjamin Kramer, Jacob Nattermann, Ulrich Spengler, Christian P. Strassburg, David H. Adams, Ye H. Oo

**Affiliations:** ^1^National Institute of Health Research Liver Biomedical Research Unit Birmingham, Centre for Liver Research, Institute of Immunology and Immunotherapy, University of Birmingham, Birmingham, United Kingdom; ^2^Department of Internal Medicine I, University of Bonn, Bonn, Germany; ^3^German Center for Infection Research, University of Bonn, Bonn, Germany; ^4^Swansea University Medical School, Institute of Life Science, Swansea University, Swansea, United Kingdom; ^5^Human Biomaterial Resource Centre, University of Birmingham, Birmingham, United Kingdom; ^6^University Hospital of Birmingham NHS Foundation Trust, Birmingham, United Kingdom

**Keywords:** ascites, *Escherichia coli*, liver, lymphocyte, NK cells, peritoneal cavity, spontaneous bacterial peritonitis

## Abstract

**Background and Aims:** Ascites and spontaneous bacterial peritonitis (SBP) are frequent complications of liver cirrhosis. In spite of the clinical impact, knowledge about ascites as an immune cell compartment in liver disease is limited. Therefore, we analyzed NK cells in blood, ascites, and liver.

**Methods:** Mononuclear cells from blood, ascites, and liver explants of patients with advanced liver disease were extracted by density gradient centrifugation. Phenotyping and analysis of functional responses were carried out using flow cytometry. Migratory potential was investigated with transwell chamber assays. NK cell metabolism was assessed by Seahorse technology.

**Results:** NK cell frequency was increased in uninfected ascites compared to blood, but not to liver. Ascites NK cells were predominantly CD16^positive^. CD56^bright^ ascites NK cells did not share the typical phenotype of their liver counterparts. In contrast to the inhibitory receptor NKG2A, expression of the activating receptor NKG2D was decreased on ascites and liver CD16^positive^ NK cells. Ascites NK cells expressed higher levels of CXCR3 than blood or liver NK cells, corresponding to increased ascites levels of CXCL10. Blood NK cells migrated toward ascites. Stimulation of mononuclear cells with *Escherichia coli* led to downregulation of NKG2D expression and IL-12 and IL-18 mediated secretion of interferon-γ by ascites and liver, but not blood NK cells. *In-vivo*, ascites NK cells expressed higher levels of the activation marker CD69 and lower levels of NKG2D during SBP compared to uninfected ascites.

**Conclusion:** Ascites NK cells display a particular phenotype and are implicated in local immune defense against translocating bacteria.

## Introduction

Despite advances in medical treatment, bacterial peritonitis is still a life-threatening infection. This applies in particular to patients with liver cirrhosis, who are highly susceptible to spontaneous bacterial peritonitis (SBP) caused by bacterial translocation from the intestine (1). In cirrhotic patients, the peritoneal cavity is therefore not only an immunological compartment of interest, but also accessible for research, because these patients often develop ascites and require paracentesis. Ascites, which contains peritoneal immune cells, comprises mainly monocytes/macrophages, which are seen as primary line of defense against bacteria, and lymphocytes (2). Neutrophil numbers rise only during bacterial peritonitis to significant levels, which makes a neutrophil count >250 cells/μL a reliable diagnostic marker for bacterial peritonitis (1). Murine studies indicate that also lymphocytes, in particular NK cells, play an important role in bacterial peritonitis (3, 4). However, a potential involvement of human NK cells in peritoneal antibacterial defense has not been investigated. Taking advantage of the accessibility of NK cells from different tissues in patients with liver cirrhosis, we hypothesized that ascites NK cells may differ concerning phenotype and function from NK cells from other relevant tissues such as liver and blood and that they take part in antibacterial response.

## Patients and methods

### Patients

Patients with decompensated liver cirrhosis were included in this study if they were at least 18 years old and had given informed consent. Diagnosis of liver cirrhosis was based on liver histology or on clear evidence of portal hypertension by clinical, laboratory, and radiological findings in presence of chronic liver disease. When patients received a diagnostic or therapeutic paracentesis, ascites, and blood were collected. In patients undergoing liver transplant, a slice of explanted liver tissue was processed as described below. Paracentesis was performed and SBP was diagnosed according to international guidelines (1). Demographical, clinical, and standard laboratory data of the patients were retrieved from the medical records. The study was approved by the local ethics committee (HBRC 16-261).

Two cohorts of patients were analyzed (Table 1). In a first cohort (tissue cohort), liver, ascites and blood samples from patients without SBP (*n* = 43) were collected to investigate differences between these tissues. To assess the impact of SBP on NK cell phenotype, ascites samples with (*n* = 8) and without SBP (*n* = 15) from a second cohort (SBP cohort) were compared. Samples are from patients without SBP unless otherwise stated.

**Table 1:** Patient characteristics.

	**Tissue cohort**	**SBP cohort**	
		**Without SBP**	**With SBP**
Patients [*n*]	43	15	8
SBP samples [*n*]	0	0	8
Male sex [*n*, %]	32 (74%)	10 (67%)	5 (63%)
Age [years]	53 (45; 65)	54 (56; 67)	67 (57; 71)
Etiology of liver cirrhosis [*n*, %]			
- Alcohol	22 (51%)	11 (73%)	3 (37%)
- NAFLD	4 (9%)	–	–
- PBC	4 (9%)	–	–
- PSC	6 (14%)	–	–
- Cryptogenic	1 (2%)	2 (13%)	1 (13%)
- Other^*^	6 (14%)	2 (13%)	4 (50%)
MELD score [points]	14 (12; 18)	14 (12; 18)	21 (19; 28)
Serum albumin [g/L]	33 (31; 37)	29 (26; 34)	29 (23; 33)
Ascites PMN [/μL]	40 (9; 95)	36 (9; 52)	847 (264; 3953)
Ascites total protein [g/L]	19 (11; 22)	11 (6; 18)	11 (5; 16)
Culture positive ascites	0	0	5 (63%)
Serum LBP [μg/mL]	2.4 (1.0; 3.3)	–	–
Ascites samples [*n*]	18	15	8
Blood samples [*n*]	15	–	–
Liver samples [*n*]	26	–	–

*Data are given as absolute numbers [%] or median (interquartiles). MELD, model for end-stage liver disease; NAFLD, non-alcoholic fatty liver disease; PBS, primary biliary cholangitis; PSC, primary sclerosing cholangitis*.

* *including α1-antitrypsin deficiency, hemochromatosis, toxic, vascular, Wilsons's disease*.

### Methods

#### Isolation of Peripheral Blood Mononuclear Cells (PMBC), Ascites Mononuclear Cells (AMC), and Liver Infiltrating Mononuclear Cells (LMC)

Venous blood was collected in EDTA tubes. Up to 1l of ascites was collected in sterile culture flasks. PBMC and LMC were isolated as described previously (5). After centrifuging ascites for 10 min at 2,000 rpm, the cell pellet was resuspended in an appropriate volume of ascites supernatant. AMC were separated by density gradient centrifugation using Lympholyte (Cedarlane) or Ficoll-Paque (Biochrom AG, Berlin, Germany). Cells from the tissue cohort were washed twice in PBS, counted and used immediately for experiments comparing different tissues. Cells from the SBP cohort were stored at −150°C until analysis. Because, as expected in research on different kinds of human diseased tissue, the number of cells available for experiments differed between tissues and donors, it was not possible to provide equal numbers of staining/experiments for all three tissues and all markers.

#### Analysis of Mononuclear Cells From Different Tissues by Flow Cytometry

Expression of surface and intracellular markers was analyzed by flow cytometry. Briefly, cells were stained with e506 viability dye (eBiosience) for later exclusion of dead cells for 30 min at 4°C in PBS. After washing with 2% fetal calf serum (FCS, Sigma-Aldrich), cells were incubated on ice in the presence of antibodies against the respective surface markers at the appropriate dilution in 2% FCS for 30 min. After washing with 2% FCS, the cells were either fixed for 10 min in 3% formaldehyde solution (Sigma-Aldrich) or fixed and permeabilised for 45 min at 4°C with the Foxp3/Transcription Factor Staining Buffer Set (eBioscience) to allow staining for intracellular antigens. Together with appropriate compensation controls (anti-mouse IgGκ /negative control compensation particles, BD Biosciences), data were acquired on a CyAN ADP or BD Canto II flow cytometer. Details on the antibodies used in this study are given in Supplementary Table 1. NK cells were identified as viable, single cells within the lymphocyte region in the forward/side scatter plot expressing CD56, but not CD3 (general gating strategy and exemplary representative dots plots in Supplementary Figure 1).

#### Enrichment of NK Cells

NK cells were magnetically enriched using the EasySep^TM^ Human NK Cell Isolation Kit (Stemcell Technologies) according to the protocol provided by the manufacturer. NK cell purity > 90% was achieved.

#### Cell Stimulation With *Escherichia coli*

*E. coli* DH5α (Invitrogen) were grown in LB broth overnight, washed twice in sterile PBS, fixed with 2% formaldehyde solution for 30 min and washed again twice in sterile PBS (6). For cell stimulation experiments, 0.5 × 10^6^ mononuclear cells in RPMI-1640 medium containing penicillin (100 IU/mL), streptomycin (100 IU/mL), glutamine (2 mM) (GIBCO, Carlsbad,CA, USA), and 10% FCS were incubated at a 1:10 ratio with fixed bacteria for 18 h in a 24 well plate at 37°C and 5% CO_2_-in-air. For analysis of cytokine production, brefeldin A was added at a final concentration of 5 μg/mL for the last 4 h of incubation. Finally, the cells were collected and stained as indicated above for flow cytometry. Intracellular staining was carried out after fixation with 3% formaldehyde solution by incubating the cells in 0.1% saponin solution containing the antibodies of interest for 30 min. For some functional experiments, blocking antibodies or the appropriate isotype controls were added, using the following antibodies: anti-IL12p70 (clone #24910, R&D), anti-IL18 (clone 125-2H, MBL), and anti-IFN-γ (clone B27, Biolegend) at final concentrations of 5 μg/mL, 5 μg/mL, and 10 μ/mL, respectively.

#### Cell Migration Experiments

Wells were prepared with RPMI-1640 medium as a negative control, ascites supernatant, or plasma. PBMC isolated from haemochromatosis patients, who are suitable donors for control PBMC because these patients have to undergo therapeutic phlebotomy on a regular basis, but are in stable condition, were added into the top chamber of 3 μm transwell inserts (Corning, Sigma-Aldrich) in RPMI. In some experiments, PBMC were pre-incubated with a CXCR3 blocking antibody (clone G025H7, Biolegend) at 10 μg/mL, an appropriate isotype control or pertussis toxin (100 ng/mL) for 30 min. The plates were incubated for 4 h at 37°C and 5% CO_2_-in-air. Then, the fluid in the lower chamber was collected. Cells were stained with anti-CD3 and anti-CD56 as described above and analyzed by flow cytometry using AccuCheck counting beads (Thermo Fisher Scientific) for quantification.

#### CD107a Assay

AMC were incubated as described above at a 1:10 ratio with fixed *E. coli* in the presence of anti-CD107a antibodies (clone H4A3, BD Biosciences) for 5 h, adding GolgiStop as recommended by the manufacturer (BD Biosciences) after the first hour. After staining, cells were then analyzed by flow cytometry.

#### Analysis of NK Cell Metabolism

Extracellular flux analysis of purified NK cells was performed using the Seahorse XF analyzer (Agilent). Cells were initially resuspended in XF assay media (Agilent) supplemented with 5.5 mM glucose and 1 mM pyruvate. 2 × 10^5^ NK cells were seeded onto a Cell-Tak (Corning) coated microplate. The oxygen consumption rate (OCR; pmoles/min) was measured during the mitochondrial stress assay with use of real-time injections; oligomycin (1 μM), carbonyl cyanide-*4*-(trifluoromethoxy) phenylhydrazone (FCCP; 1 μM) and rotenone/antimycin A (both 1 μM) (both from Sigma-Aldrich).

#### LBP ELISA and Luminex Assay

Blood or ascites were collected in serum tubes, centrifuged for 10 min at 1,300 × g and supernatant was stored in aliquots at −80°C. LBP levels were determined using the human LBP Duoset ELISA kit from R&D. In 8 matched pairs of serum and ascites from the same patients, 27 cytokines were measured by the Bio-Plex ProTM Human Cytokine 27-plex assay (Bio-Rad). Both assays were used according to the instructions given by the manufacturer.

#### Analysis of Data and Statistical Analysis

Flow cytometry data were analyzed with FlowJo (Tree Star). Prism 7 (Graphpad) was used for graphical presentation of the data. Statistical analyses were performed with SPSS (IBM) version 22. Fisher's exact test, Mann-Whitney-*U*-test, Wilcoxon signed-rank test, and Kruskal-Wallis test with Bonferroni correction were used as appropriate.

## Results

### NK Cell Frequency Is Increased in Ascites

**Figure 1:**
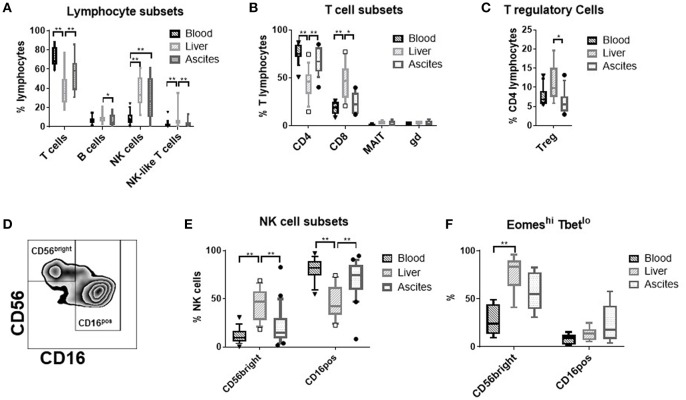
Frequency of lymphocyte-subsets in different tissues. **(A)** T cells (CD3^+^); B cells (CD19^+^), NK cells (CD3^−^CD56^+^), and NK-like T-cells (CD3^+^CD56^+^) (*n* = 9–21); **(B)** T cell subsets: CD4 T cells (CD3^+^CD4^+^), CD8 T Cells (CD3^+^CD8^+^) (*n* = 11–18); mucosal associated invariant T (MAIT) cells (CD3^+^CD161^++^TCR Vα7.2^+^), γδ T-cells (CD3^+^TCR γδ^+^) (*n* = 3–4); **(C)** T regulatory (reg) cells (CD3^+^CD4^+^CD25^high^CD127^low^) (*n* = 9–13); **(D)** representative flow cytometry plot showing the gating of the NK cell subsets; **(E)** frequency of the major NK cell subsets CD56^bright^CD16^negative^ vs. CD16^positive^ (*n* = 16–21); **(F)** frequency of the Eomes^hi^Tbet^lo^ phenotype (*n* = 6–10); *p < 0.05; **p < 0.005.

Comparing the relative frequency of T-, B-, NK-, and NK-like T-cells (Figure 1A), of CD4-, CD8-, mucosal invariant T-cells (MAIT), γδ T-cells (Figure 1B), and T regulatory cells (Figure 1C) between ascites, peripheral blood and liver, we detected a significantly higher proportion of NK cells among lymphocytes in ascites and liver compared to blood. Therefore, we focused our further analysis on NK cells.

### Ascites NK Cells Are Phenotypically Different

CD56^bright^CD16^negative^ vs. CD16^positive^ NK cells constitute the main NK cell subsets (Figure 1D). Ascites NK cells were predominantly CD16^positive^ (Figure 1E). CD56^bright^ NK cells from the liver express the transcription factor Eomes, but not Tbet (7). This phenotype was of intermediate frequency in ascites compared to liver and blood (Figure 1F).

**Figure 2:**
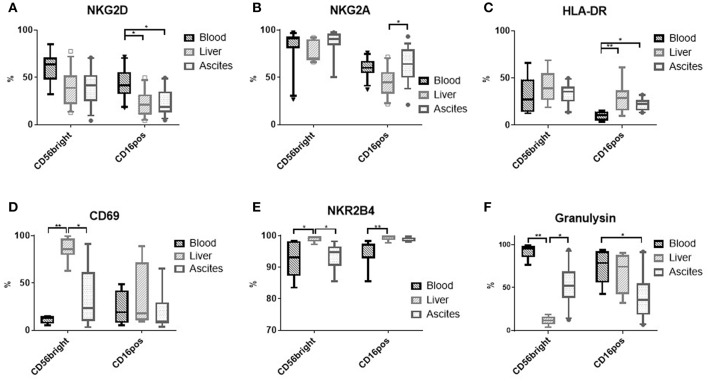
Phenotype of NK cells from different tissues. **(A)** NKG2D (*n* = 9–13); **(B)** NKG2A (*n* = 10–12); **(C)** HLA-DR (*n* = 7–10); **(D)** CD69 (*n* = 4–9); **(E)** NKR2B4 (*n* = 6 each); **(F)** Granulysin (*n* = 7–10); *p < 0.05; **p < 0.005.

Comparing typical NK cells markers, we found that NK cells from ascites show a particular expression pattern compared to liver and blood. While CD16^positive^ NK cells from ascites and liver expressed less of the activating receptor NKG2D in comparison to blood CD16^positive^ NK cells (Figure 2A and Supplementary Figure 2A), the inhibitory receptor NKG2A was found more frequently on ascites than liver CD16^positive^ NK cells (Figure 2B). A higher degree of activation in CD16^positive^ NK cells from ascites and liver was indicated by increased HLA-DR expression compared to blood (Figure 2C and Supplementary Figure 2B). With respect to expression of CD69 (Figure 2D) and the activating receptor NKR2B4 (Figure 2E), CD56^bright^ NK cells from the ascites and blood showed comparably low levels of expression, in contrast to the liver. The cytotoxic molecule granulysin was lower in ascites and liver NK cells compared to blood for both NK cell subsets, with liver CD56^bright^ NK cells displaying the lowest expression (Figure 2F). Expression of NKp46 did not differ between the tissues and the maturation marker CD27 was expressed at comparatively low levels among NK cells, with only liver CD56^bright^ NK cells tending to display higher expression levels (data not shown). Analysis of mean fluorescence intensity (MFI) levels of these markers led to similar results (Supplementary Figure 3). Taken together, subset composition and expression of typical markers is different in NK cells from ascites compared to liver and blood.

### Ascites NK Cells Express CD103 and High Levels of CXCR3

**Figure 3:**
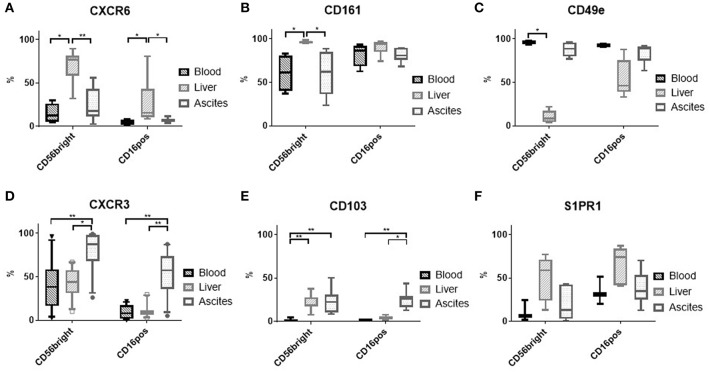
Expression of tissue homing receptors on NK cells. **(A)** CXCR6 (*n* = 4–7); **(B)** CD161 (*n* = 4–6); **(C)** CD49e (*n* = 2–5); **(D)** CXCR3 (*n* = 12–14); **(E)** CD103 (*n* = 6–7); **(F)** S1PR1 (*n* = 3–5); *p < 0.05; **p < 0.005.

**Figure 4:**
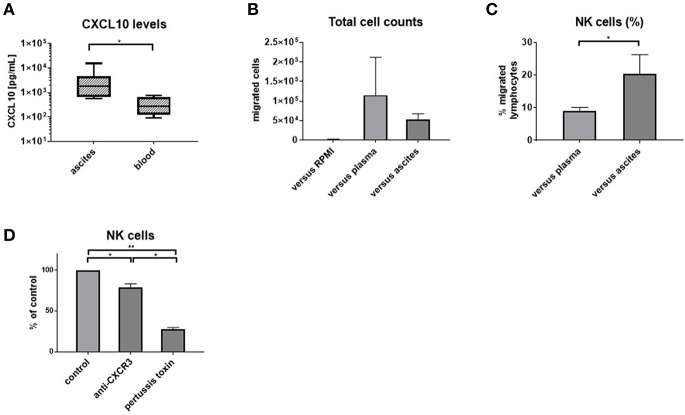
Ascites is chemotactic for NK cells. CXCL10 levels in matched ascites and blood (*n* = 8) **(A)** and results of transwell migration assays: **(B)** absolute numbers of migrated cells (*n* = 5) **(C)** blood NK cells are enriched among cells migrating toward ascites (*n* = 5) **(D)** migration is dependent on CXCR3 and G-protein-coupled signaling, with bars showing the reduction of NK cell migration compared to the respective control of PBMC migrating vs. ascites in each experiment *p < 0.05, **p < 0.005.

Because proliferation of NK cells was similar in the three compartments (Supplementary Figure 4), we analyzed tissue homing markers on NK cells to understand the higher frequency seen in ascites. High expression of CXCR6 (Figure 3A) and CD161 (Figure 3B), but low expression of CD49e (Figure 3C) have been reported for liver CD56^bright^ NK cells in contrast to blood NK cells (8, 9), but were not found in ascites. The chemokine receptor CXCR3, though, was observed more frequently on ascites NK cells compared to liver and blood (Figure 3D and Supplementary Figure 2C). In addition, expression of the integrin CD103 was increased on ascites CD16^positive^ NK Cells (Figure 3E and Supplementary Figure 2D). Sphingosine 1 phosphate receptor (S1PR) subtype 5 has been suggested as a homing marker on CD16^positive^ NK cells (10), but was not detected on NK cells from any of the three tissues (data not shown). S1PR1 was expressed mainly on liver NK cells (Figure 3F). Analysis of MFI levels of these homing markers revealed similar results (Supplementary Figure 5). Corresponding to the increased CXCR3 expression levels of ascites NK cells, we found increased levels of its ligand CXCL10 in ascites compared to matched blood (Figure 4A). These results suggest that enrichment of CD16^positive^ NK cells in ascites may be related to homing integrin CD103 and chemokine receptor CXCR3.

### NK Cells Migrate Preferentially Toward Ascites

To corroborate our phenotypic findings, we performed migration experiments with control PBMC from hemochromatosis patients vs. ascites compared to plasma. As expected, more cells migrated vs. plasma or ascites than medium (Figure 4B). However, NK cells were enriched among PBMC that migrated vs. ascites compared to plasma (Figure 4C). Blocking CXCR3 before the migration assay reduced the number of NK cells migrating toward ascites (Figure 4D), suggesting that the CXCR3-CXCL10 axis is involved in the migration of NK cells to the ascites. However, pertussis toxin, which blocks G-protein coupled signaling, inhibited migration even more, indicating that other chemokines play an additional role.

### Ascites NK Cells Participate in the Immune Response to *Escherichia coli*

**Figure 5:**
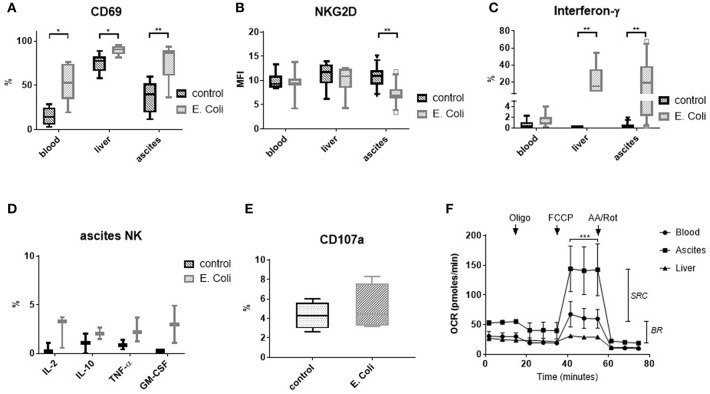
Response of NK cells to stimulation with *E. coli* and metabolic capacity of NK cells. Mononuclear cells from different tissues were stimulated with formaldehyde-fixed *Escherichia coli* (*E. coli*) **(A–E)**. Metabolic capacity of purified NK cells was measured by Seahorse-technology. **(A)** CD69 expression (*n* = 6–8) **(B)** NKG2D expression (*n* = 6–12) **(C)** Interferon-γ production (*n* = 6–11) **(D)** Production of other cytokines/chemokines by ascites NK cells (*n* = 2–3) **(E)** CD107 expression (*n* = 4) **(F)** Mitochondrial stress assay measuring the oxygen consumption rate (OCR) of purified NK cells (*n* = 3 for ascites/blood; *n* = 1 for liver). The drops/rises in OCR reflect the successive injection of oligomycin (1 μM), FCCP (1 μM), and antimycin A/rotenone (AA/Rot) (both 1 μM). *p < 0.05; **p < 0.005; ***p < 0.001; BR, basal respiration; GM-CSF, granulocytes-macrophage colony-stimulating factor; IFN-γ, interferon-γ; IL, interleukin; MFI, mean fluorescence intensity; OCR, oxygen consumption rate; oligo, oligomycin; SRC, spare respiratory capacity; TNF-α, tumor necrosis factor-α.

In the context of bacterial translocation occurring in patients with liver cirrhosis, immune cells in the peritoneal cavity are in direct contact with intestinal bacteria and bacterial products. Therefore, we stimulated mononuclear cells from ascites, blood and liver with *Escherichia coli* (*E. coli*). *E. coli* stimulation led to upregulation of CD69 expression in NK cells from all tissues (Figure 5A). In contrast, NKG2D expression was downregulated only in ascites NK cells (Figure 5B). Interferon-γ was produced upon stimulation with *E. coli* by significant numbers of NK cells from ascites and liver, but not from blood (Figure 5C and Supplementary Figures 2E,F). In addition, few ascites NK cells secreted interleukin-2 (IL-2), interleukin-10 (IL-10), tumor necrosis factor-α (TNF-α), and granulocytes-macrophage colony-stimulating factor (GM-CSF) in response to *E. coli* (Figure 5D). We considered that *E. coli* stimulated monocytes/macrophages might elicit a cytotoxic NK cell response as described previously (11). However, only low CD107a expression on NK cells after exposure to *E. coli* stimulated monocytes/macrophages was detected (Figure 5E), suggesting a different role for ascites NK cells. When comparing CD56^bright^ to CD16^positive^ NK cells, comparable effects of stimulation with *E. coli* were detected, with CD56^bright^ NK cells producing cytokines at higher frequency than CD16^positive^ NK cells (Supplementary Figures 6, 7).

### Ascites NK Cells Display an Increased Metabolic Capacity

To assess the metabolic capacity of ascites NK cells, we measured their mitochondrial function using seahorse technology. In this assay, selective inhibitors are successively added and changes in oxygen consumption are assessed. Interestingly, ascites NK cells were found to have similar basal respiration to matched blood NK cells, but a significantly higher spare respiratory capacity (Figure 5F).

### Interferon-γ Secretion by Ascites NK Cells Is Dependent on Interleukin-12 and Interleukin-18

**Figure 6:**
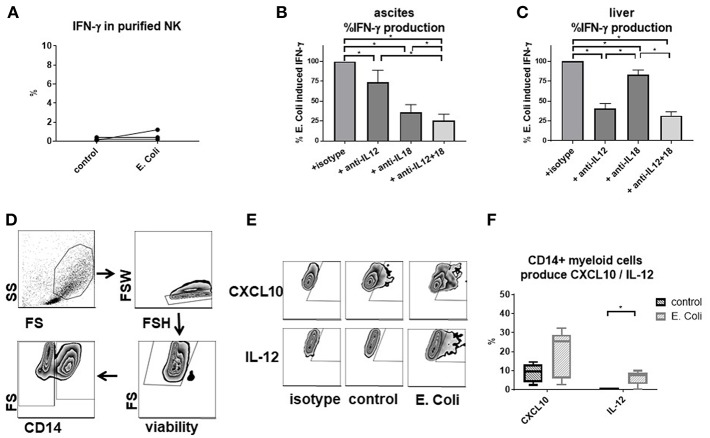
*E. coli*-induced interferon-γ production by NK cells is mediated by cytokines secreted by CD14^positive^ myeloid cells. Analysis of factors required for interferon-γ response by NK cells to *Escherichia coli* (*E. coli)* and assessment of CD14^positive^ myeloid cells as producers of relevant cytokines by flow cytometry. **(A)** stimulation of purified ascites NK cells with *E. coli* (*n* = 3); effect of blocking interleukin-12 and interleukin-18 on interferon-γ production by NK cells after *E. coli* stimulation of mononuclear cells from ascites **(B)** or liver **(C)** (*n* = 6 each) **(D)** gating strategy for ascites CD14^positive^ myeloid cells **(E)** representative zebra plots of CXCL10 and IL-12 staining **(F)** production of CXCL10 and interleukin-12 in CD14^positive^ ascites myeloid cells (*n* = 5); *p < 0.05.

Direct stimulation of purified NK cells with *E. coli* failed to elicit an IFN-γ response (Figure 6A), suggesting that mediators secreted by other immune cells might be required. By adding blocking antibodies, we observed that IFN-γ secretion is mediated mainly by IL-12 and IL-18, with IL-18 and IL-12 being the main inducer in ascites and liver, respectively (Figures 6B,C). We wondered whether ascites myeloid CD14^positive^ cells might contribute to NK cell migration and function in the peritoneal cavity. In support of this hypothesis, ascites myeloid CD14^positive^ cells produced CXCL10, the ligand for CXCR3, and, after *E. coli* stimulation, IL-12 (Figures 6D–F).

### Ascites NK Cells Are Activated During SBP *in vivo*

**Figure 7:**
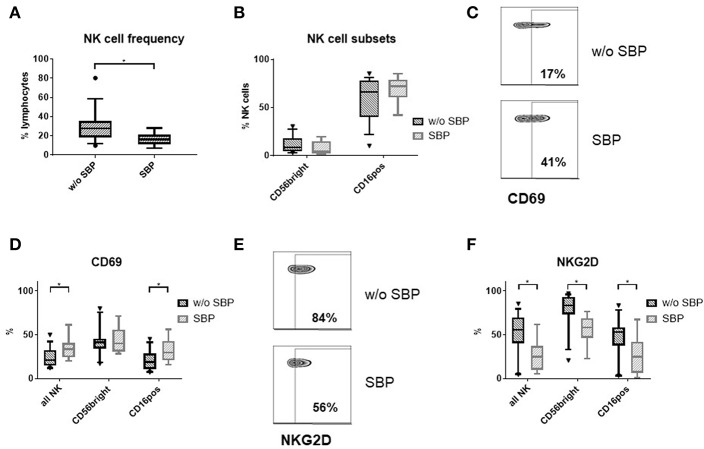
Ascites NK cell phenotype in patients with SBP. **(A)** NK cell frequency among lymphocytes in the ascites of patients with (*n* = 8) and without spontaneous bacterial peritonitis (SBP) (*n* = 15) **(B)** Ascites NK subset distribution **(C)** representative zebra plots of CD69 expression **(D)** CD69 expression of ascites NK cells **(E)** representative zebra plots of NKG2D expression on CD56^bright^ ascites NK cells **(F)** NKG2D expression of ascites NK cells; *p < 0.05.

To ascertain that our functional *ex vivo* findings replicate the situation *in vivo*, we analyzed NK cells in ascites samples from cirrhosis patients with (*n* = 8) and without SBP (*n* = 15). NK cell frequency was decreased in patients with SBP compared to patients without SBP (Figure 7A). The proportion of CD16^positive^ and CD56^bright^CD16^negative^ NK cells did not change during SBP (Figure 7B). While the level of CD69 expression increased on total NK cells during SBP due to higher expression on CD16^positive^ NK cells (Figures 7C,D), NKG2D expression decreased on both NK cell subsets during SBP (Figures 7E,F).

## Discussion

The peritoneal cavity is an immunological compartment of particular interest. Human immune cells from the peritoneal cavity become accessible for research when patients develop ascites, which is common in patients with liver cirrhosis, in patients with chronic kidney failure undergoing peritoneal dialysis and in malignant disease such as ovarian cancer.

Studies on ascites immune cells in liver cirrhosis have been mainly performed on the role of macrophages as primary line of defense against invading bacteria (12–14). HLA-DR expression on peritoneal monocytes/macrophages was found to correlate inversely with local bacterial DNA burden, linking ascites immune cell phenotype to bacterial translocation (2). Concerning T lymphocytes, a decrease in the CD4/CD8 T-cell ratio and an increase in γδ T-cells during bacterial ascites infection have been reported (15). However, detailed reports on ascites NK cells have been lacking so far.

In patients on peritoneal dialysis, the focus of research has been peritoneal immunity in the context of peritonitis, which is frequent in patients on peritoneal dialysis (16). In these patients, a robust interferon (IFN) gamma production by peritoneal lymphocytes seems to protect against peritonitis by supporting antibacterial function of peritoneal macrophages (17). More recently, Zhang and colleagues discovered that the profile of immune cell composition and of cytokine levels in the peritoneal cavity during peritonitis is linked to the type of causative bacteria (18).

Concerning malignant ascites, most studies have been performed in ovarian cancer. Because specific subsets of tumor infiltrating lymphocytes such as cytotoxic CD8 lymphocytes or NK cells are supposed to suppress tumor growth, Bamias and co-authors investigated the relation between lymphocyte subsets and tumor grade. They detected that NKT-like cells were less frequent in the ascites from patients with cancer resistant to chemotherapy (19). Later, NK cells were found to be enriched in malignant ascites relative to blood, where they seemed to be protective (20, 21). Degranulation of NK cells from ascites of ovarian cancer patients was lower when malignant cells could be detected, indicating not only a quantitative, but also a functional relationship between NK cells and cancer progression (22). These results were confirmed when NK cells were found to be enriched and more activated in the ascites of patients with benign and malignant ovarian disease compared to blood, but less activated in malignant disease compared to benign conditions (23). This prompted a successful experimental attempt to expand autologous NK cells from ascites of patients with ovarian cancer and restore their activity *in-vitro* (24).

NK cells are well-known for their anti-viral and anti-tumor properties, for their ability to secrete cytokines, most notably IFN-γ, and to regulate other immune cells, such as macrophages (25). In addition, they have been implicated in defense against extracellular bacterial (26), for example in periodontitis (27) and urinary tract infection (28). A relative abundance of NK cells is known from liver, uterus and adipose tissue (29). NK cells from these anatomic locations display a considerable diversity in phenotype and function (29). In different experimental settings, elimination of NK cells in murine peritonitis models led to lower levels of inflammatory cytokines, decreased activation of peritoneal macrophages and longer survival of mice (3, 4, 30), but a role for human NK cells during bacterial peritonitis had not been investigated. In humans, peritoneal NK cells from healthy individuals are not accessible for research. However, patients with liver cirrhosis and ascites offer the unique opportunity to study tissue NK cells in presence and absence of peritonitis.

Concerning the predominant intrahepatic NK cell population, CD56^bright^ NK cells, we found a phenotype consistent with previous observations (7–9, 31, 32): they constitute the majority of liver NK cells and express Eomes, CXCR6 and CD69, but not Tbet, CD49e or cytotoxic granules. In ascites, by contrast, we found that CD16^positive^ NK cells predominate, which do not express these markers at comparable levels to CD56^bright^ hepatic NK cells. However, ascites NK cells displayed higher expression levels of the chemokine receptor CXCR3 compared to liver and blood. Correspondingly, we confirmed a previous report that ascites contains elevated levels of the CXCR3 ligand CXCL10 (33), and found that CXCL10 is produced by local CD14^positive^ myeloid cells. Trafficking of NK cells into the peritoneal cavity via CXCR3 has been described in mice (4). In line, comparable levels of proliferation among NK cells from all three compartments indicated that migration may contribute to the increased NK cells frequency in ascites. In further support of this hypothesis, transwell experiments showed an enhanced migration of human NK cells toward ascites compared to plasma, which was mediated by CXCR3 and G-protein-coupled signaling. Furthermore, ascites NK cells expressed increased levels of CD103, whose ligand, E-cadherin, is present on human mesothelial cells (34), facilitating transmigration of CD103^positive^ NK cells in the ascites. A high expression of CD103 and CXCR3 was also found in CD56^bright^ NK cells in the ascites. The rather similar phenotype of ascites and blood NK cells together with a comparable frequency of CD16^positive^ NK cells indicate that NK cells migrate to the ascites rather from blood than from the liver.

Furthermore, ascites CD16^positive^ NK cells differed from blood NK cells by upregulated HLA-DR expression, indicating a higher level of activation. We also detected a higher spare energetic capacity, providing the energetic basis for activation. The abundant bacterial products in the peritoneal cavity in patients with liver cirrhosis (2, 35) might activate immune response and induce upregulation of HLA-DR. Similarly, stimulation of mononuclear cells with TLR ligands might explain why NKG2D expression was downregulated in ascites (36). NKG2D is critical for pulmonary clearance of Gram-negative infections (37), indicating that this receptor is particularly important in the context of NK cell immune response to bacterial infections.

*In-vitro* stimulation with *E. coli* was used as functional assay mimicking SBP, which is the clinically most important local complication of ascites formation in patients with liver cirrhosis. We chose fixation of the bacteria with formaldehyde (6) to exclude additional effects by bacterial growth. However, this fixation method may alter the antigen structures and the extracellular substance of the bacteria (38). *In-vitro* stimulation with *E. coli* supported activation and modulation of NKG2D expression of peritoneal NK cells in presence of bacteria. Interestingly, we found that the NK cell response to bacterial stimulation is dependent on tissue localization and cell-cell interactions, because *E. coli*-induced IFN-γ secretion was not elicited in blood NK cells and depended on IL-12 and IL-18 in ascites. Ascites macrophages are known to be strong producers of IL-18 upon inflammasome stimulation (39). In addition, we detected IL-12 production in CD14^positive^ myeloid ascites cells. Some features of the ascites NK cells phenotype, such as decreased NKG2D expression, would be compatible with exhaustion after chronic stimulation (40). To further corroborate our *in-vitro* findings in a clinical setting, we investigated the phenotype of ascites NK cells in patients with and without SBP. We found an activated phenotype in ascites NK cells from patients with SBP, characterized by increased CD69 and decreased NKG2D expression, indicating that NK cells take part in the local immune response during SBP.

NK cells are considered to play a dual role during bacterial infection and sepsis. They might be protective in an early stage by regulating local immune response to prevent systemic dissemination of microorganisms. At a later stage, they may drive excessive immune activation and mortality (26). Additional experiments demonstrating the impact of IFNγ on TNFα and IL-6 production by CD14^positive^ myeloid ascites suggested that NK cells might have indeed a regulatory role during peritonitis in patients with liver cirrhosis. A pro-inflammatory cytokine secretion might be particularly dangerous in patients with liver cirrhosis and SBP, who display an unbalanced immune system similar to sepsis patients (1) and whose mortality is linked to the degree of inflammatory response (41, 42). However, an association of NK cell markers to clinical events in patients with liver cirrhosis remains so far unproven. Given that our study focused on analyzing differences between tissues, we did not include a sufficiently high number of patients at risk for clinical events, such as SBP and mortality, to yield statistical power concerning clinical endpoints after correction for other critical parameters, such as severity of liver disease and antibiotic resistance. Still, we noted that in absence of SBP, expression of NKG2D on ascites CD16^positive^ NK cells was significantly lower among the 3 patients who died within 1 year compared to the 12 remaining patients (54% vs. 3%; *p* = 0.03). Follow-up studies are needed in this respect. Another interesting question would be to establish the phenotype of ascites NK cells from healthy persons. However, ascites NK cells are not accessible in healthy humans for research. In addition, in-depth mechanistic studies elucidating the detailed processes of the immune cell response in different tissues to bacteria are impossible in humans. However, the outstanding achievement of this study is to compare phenotype and function of immune cells from three different diseased human tissues.

Although the putative both protective and detrimental role of NK cells complicates approaches for therapeutic interventions, the CXCR3-CXCL10 axis might constitute a promising target. In murine peritonitis models, blockage of CXCR3 or CXCL10 in conjunction with antibiotic therapy reduced migration of NK cells to the peritoneal cavity and improved survival, even if applied after induction of peritonitis (4, 43, 44). An inhibitor of CXCL10, eldelumab, has already been tested in clinical trials for inflammatory bowel disease (45). Such a therapeutic approach might combine the benefits of damping excessive immune response without compromising the direct antibacterial function of myeloid cells. Further studies on kinetics and function of CXCR3^positive^ lymphocytes during SBP are needed to clarify if such an approach might be tested in clinical trials.

In summary, our findings indicate that peritoneal NK cells are not only relevant in malignant disease, but also in patients with liver disease, where the constitute a locally abundant cell population with a distinct phenotype and participate functionally in the inflammatory response to invading bacteria. Further research on the role of NK cells in bacterial infections in patients with liver cirrhosis might uncover new therapeutic approaches to prevent excessive immune response.

## Data Availability

All datasets generated for this study are included in the manuscript and/or the Supplementary Files.

## Ethics Statement

This study was carried out in accordance with the recommendations of Human Biomaterials Resource Center of the University of Birmingham with written informed consent from all subjects. All subjects gave written informed consent in accordance with the Declaration of Helsinki. The protocol was approved by the local ethic committee (Human Biomaterials Resource Center of the University of Birmingham, decision 16-261).

## Author Contributions

PL, JN, US, CS, DA, and YO designed the study. PL collected the samples, analyzed the data and wrote the manuscript. PL, HJ, NJ, JB, and BK performed the experiments. JN, US, CS, DA, and YO critically revised the manuscript.

### Conflict of Interest Statement

The authors declare that the research was conducted in the absence of any commercial or financial relationships that could be construed as a potential conflict of interest.
