# Supplementary material for: NK Cells in Ascites From Liver Disease Patients Display a Particular Phenotype and Take Part in Antibacterial Immune Response
Source: Front Immunol. 2019 Aug 7;10:1838. doi: 10.3389/fimmu.2019.01838 (PMC6694841; doi:10.3389/fimmu.2019.01838)
Supplement: Supplementary file 2 [file Presentation_1.PPTX]

## Slide 1
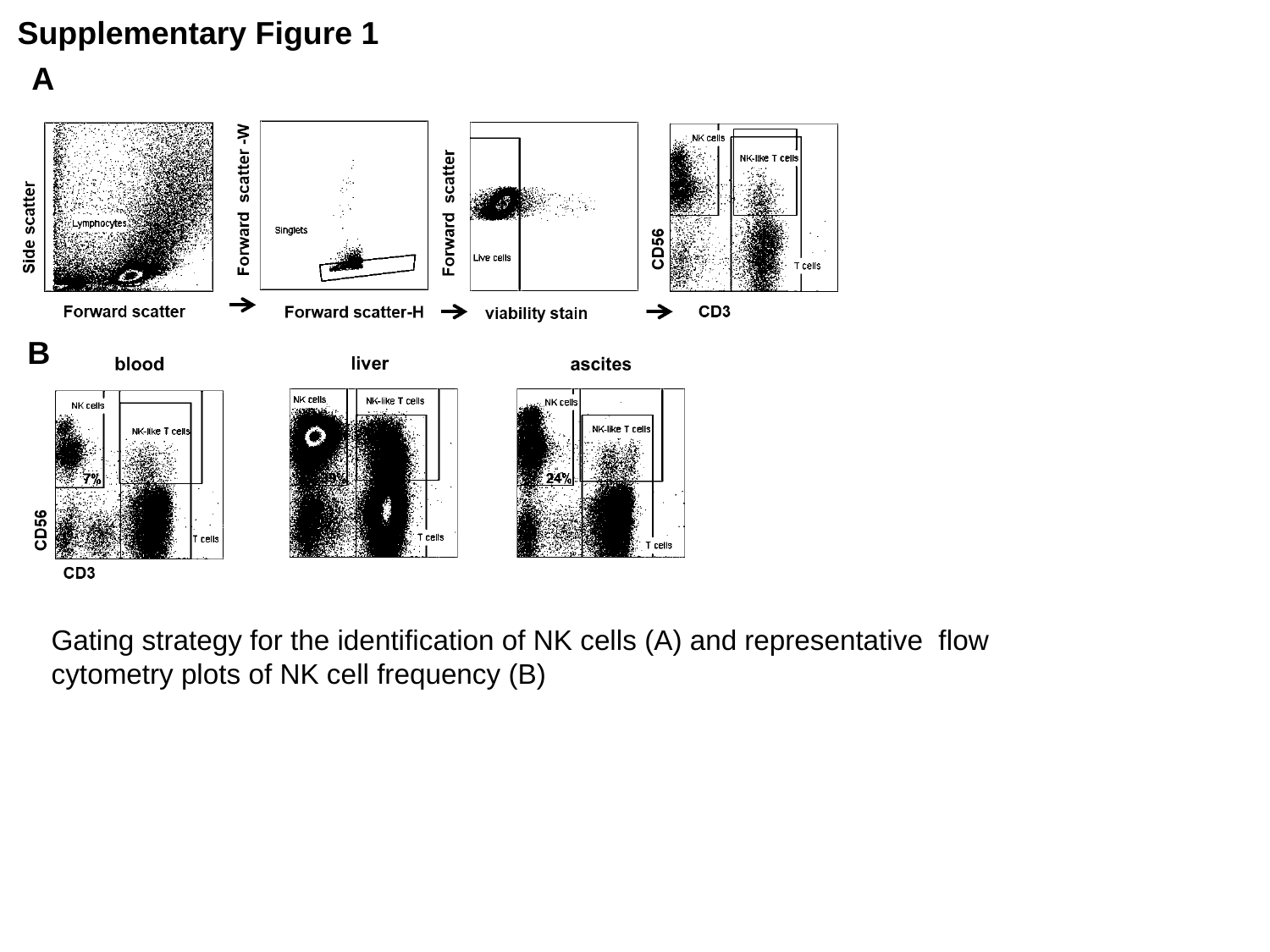

Supplementary Figure 1
A
B
Gating strategy for the identification of NK cells (A) and representative flow cytometry plots of NK cell frequency (B)

## Slide 2
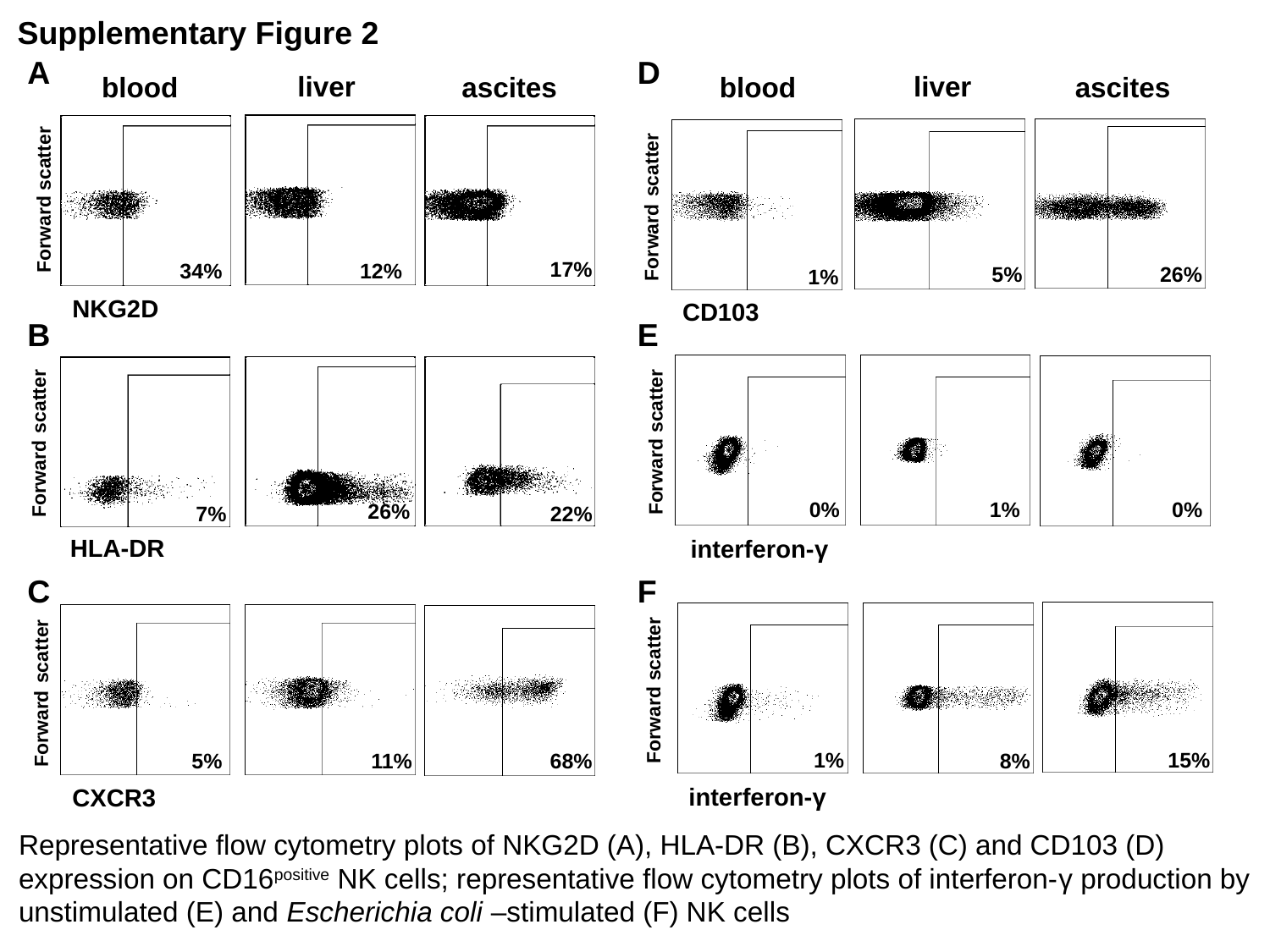

Supplementary Figure 2
A
D
liver
liver
blood
ascites
blood
ascites
Forward scatter
Forward scatter
17%
34%
12%
5%
26%
1%
NKG2D
CD103
B
E
Forward scatter
Forward scatter
1%
0%
0%
26%
22%
7%
HLA-DR
interferon-γ
C
F
Forward scatter
Forward scatter
1%
15%
5%
11%
68%
8%
interferon-γ
CXCR3
Representative flow cytometry plots of NKG2D (A), HLA-DR (B), CXCR3 (C) and CD103 (D) expression on CD16positive NK cells; representative flow cytometry plots of interferon-γ production by unstimulated (E) and Escherichia coli –stimulated (F) NK cells

## Slide 3
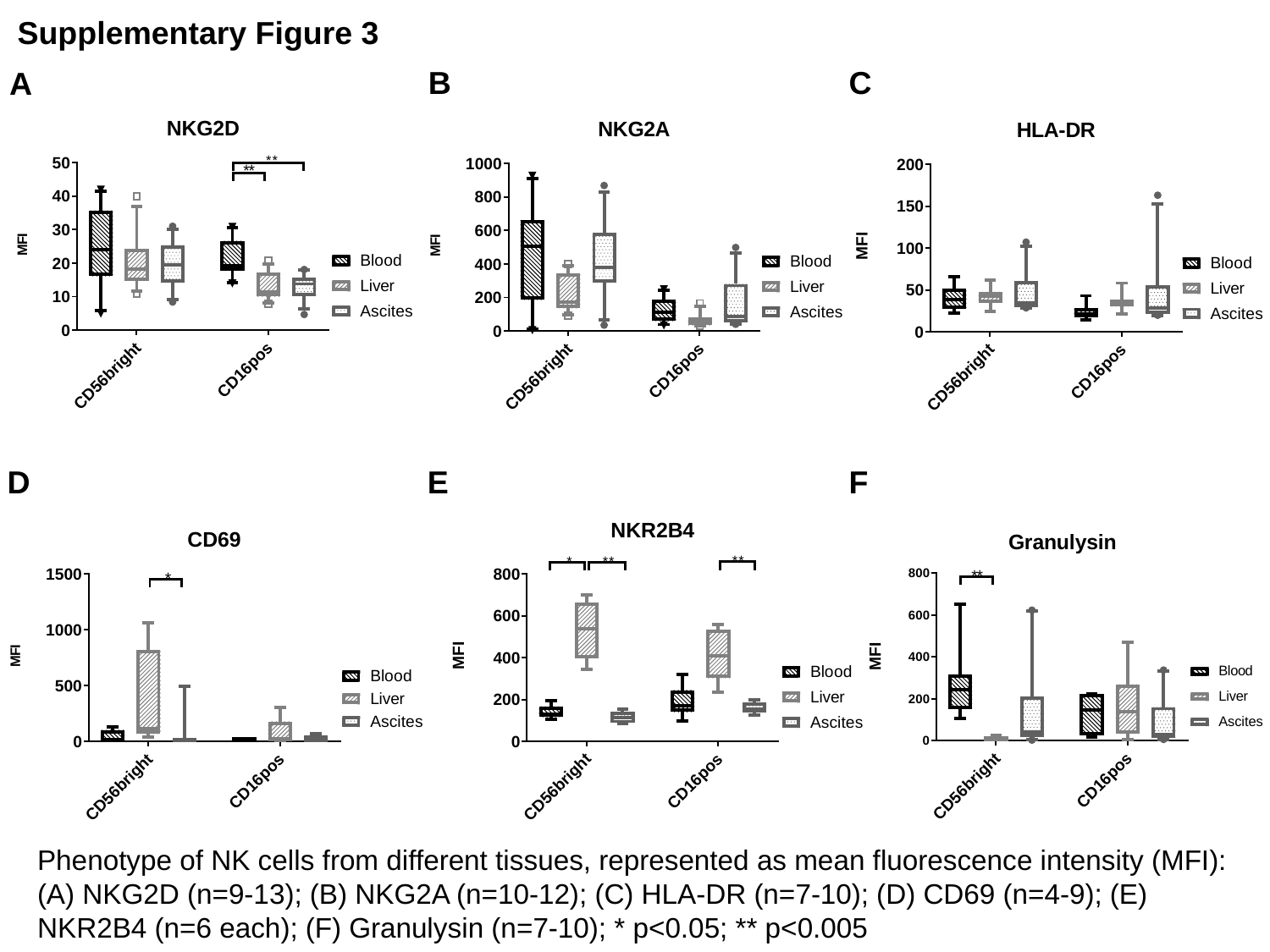

Supplementary Figure 3
B
C
A
D
E
F
Phenotype of NK cells from different tissues, represented as mean fluorescence intensity (MFI): (A) NKG2D (n=9-13); (B) NKG2A (n=10-12); (C) HLA-DR (n=7-10); (D) CD69 (n=4-9); (E) NKR2B4 (n=6 each); (F) Granulysin (n=7-10); * p<0.05; ** p<0.005

## Slide 4
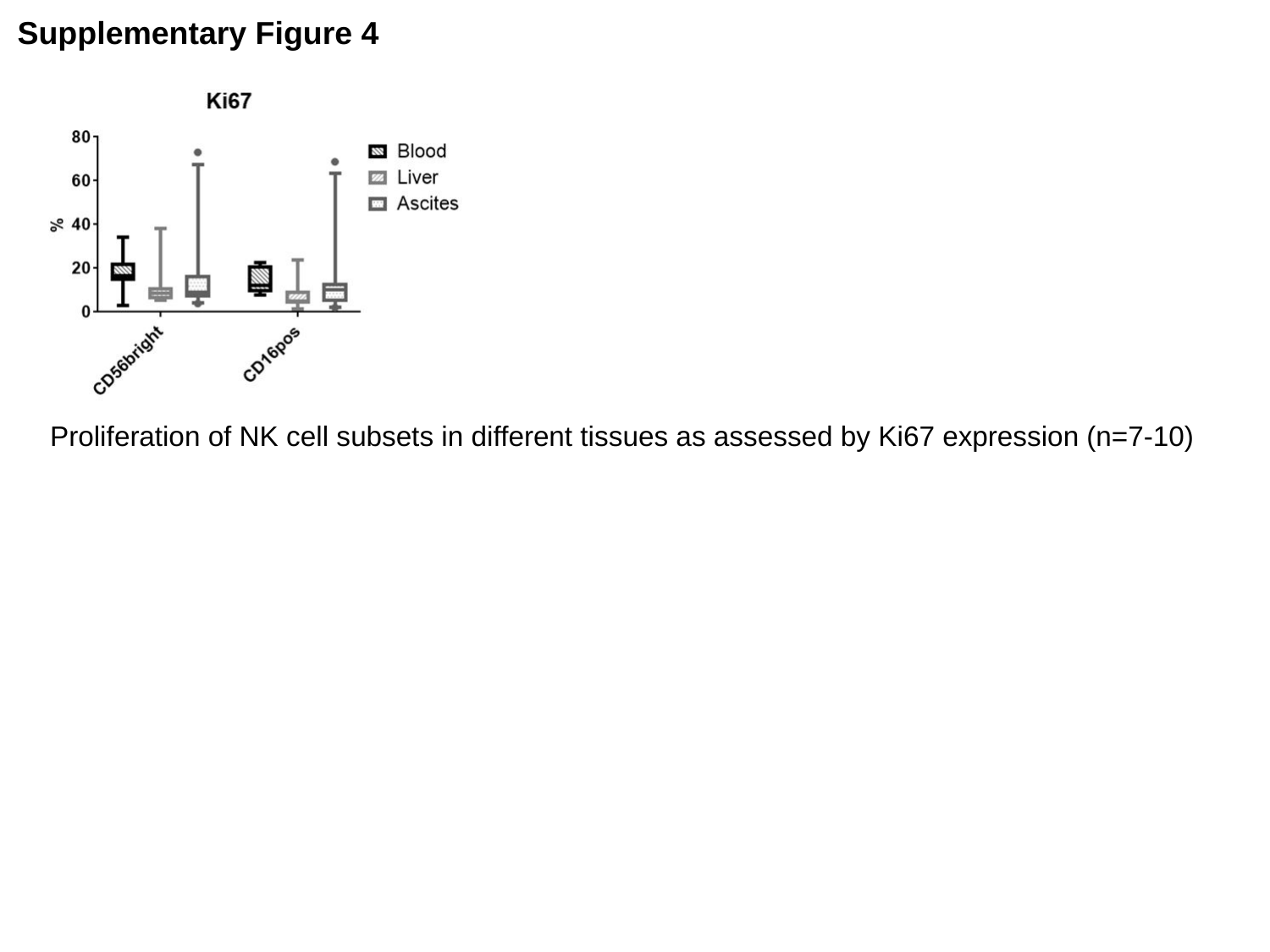

Supplementary Figure 4
Proliferation of NK cell subsets in different tissues as assessed by Ki67 expression (n=7-10)

## Slide 5
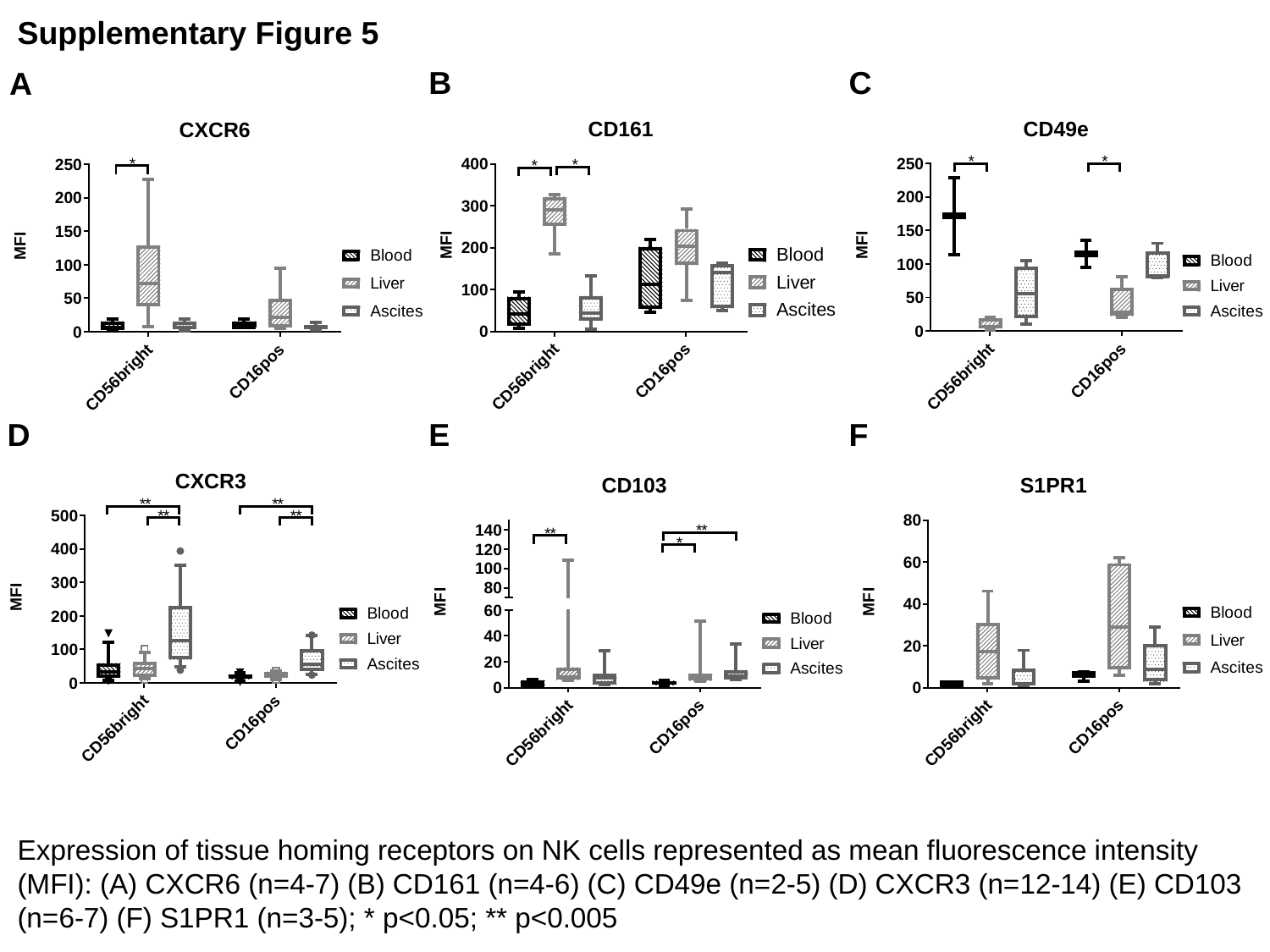

Supplementary Figure 5
B
C
A
D
E
F
Expression of tissue homing receptors on NK cells represented as mean fluorescence intensity (MFI): (A) CXCR6 (n=4-7) (B) CD161 (n=4-6) (C) CD49e (n=2-5) (D) CXCR3 (n=12-14) (E) CD103 (n=6-7) (F) S1PR1 (n=3-5); * p<0.05; ** p<0.005

## Slide 6
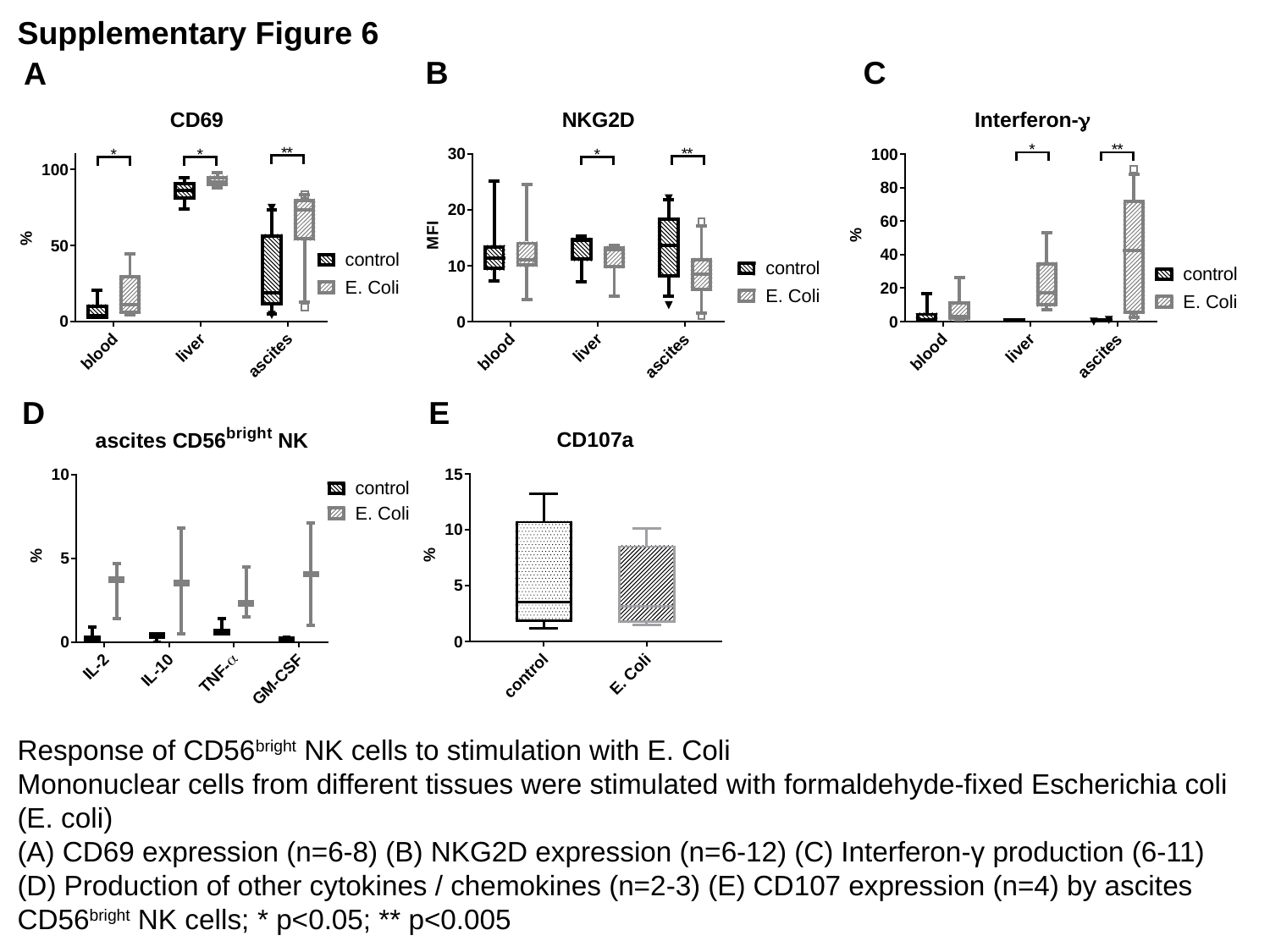

Supplementary Figure 6
B
C
A
D
E
Response of CD56bright NK cells to stimulation with E. Coli
Mononuclear cells from different tissues were stimulated with formaldehyde-fixed Escherichia coli (E. coli)
(A) CD69 expression (n=6-8) (B) NKG2D expression (n=6-12) (C) Interferon-γ production (6-11) (D) Production of other cytokines / chemokines (n=2-3) (E) CD107 expression (n=4) by ascites CD56bright NK cells; * p<0.05; ** p<0.005

## Slide 7
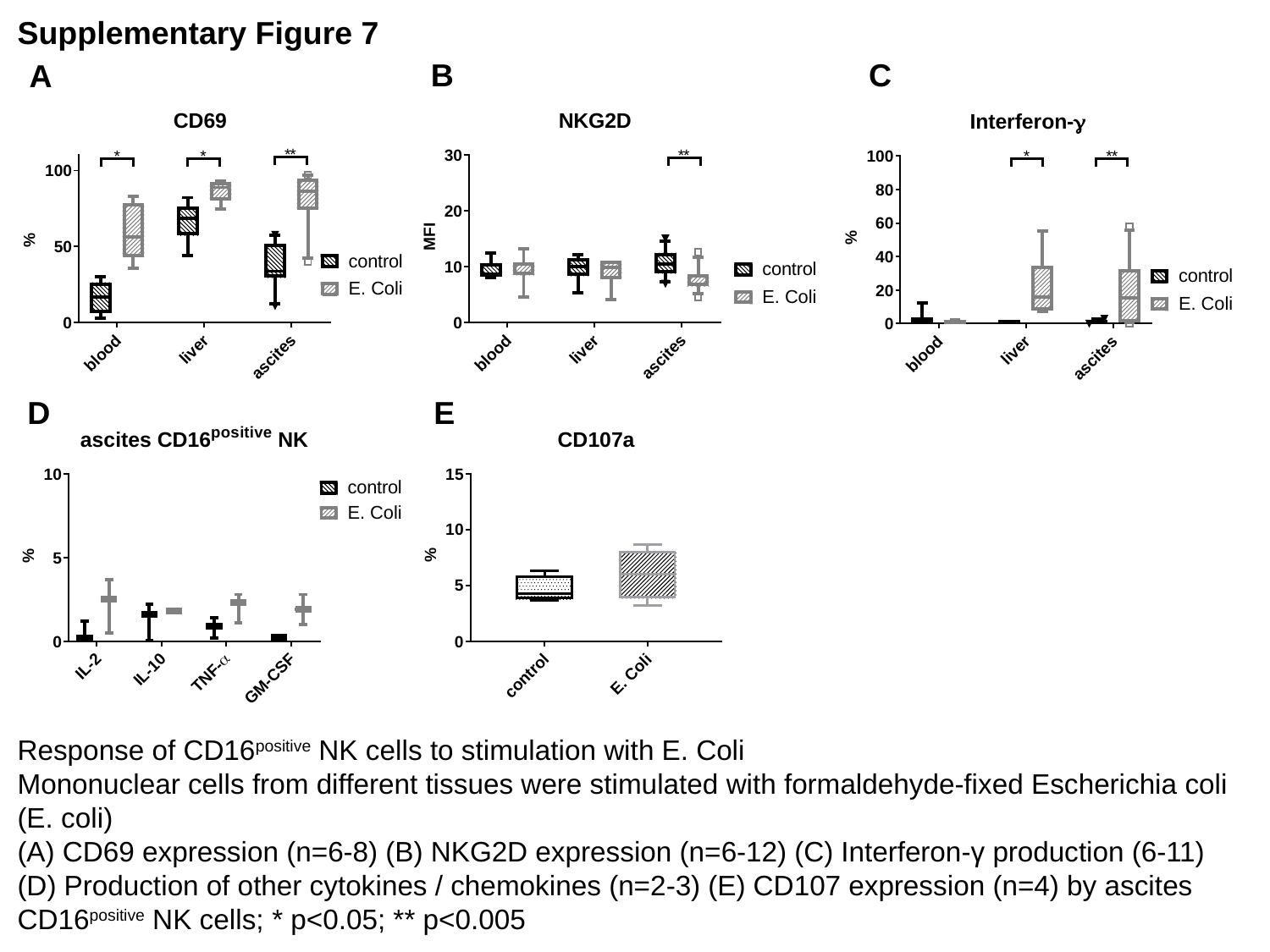

Supplementary Figure 7
B
C
A
D
E
Response of CD16positive NK cells to stimulation with E. Coli
Mononuclear cells from different tissues were stimulated with formaldehyde-fixed Escherichia coli (E. coli)
(A) CD69 expression (n=6-8) (B) NKG2D expression (n=6-12) (C) Interferon-γ production (6-11) (D) Production of other cytokines / chemokines (n=2-3) (E) CD107 expression (n=4) by ascites CD16positive NK cells; * p<0.05; ** p<0.005
